# Early continuous renal replacement therapy for acute kidney injury in a very low birth weight infant: a case report and literature review

**DOI:** 10.3389/fmed.2026.1881048

**Published:** 2026-06-18

**Authors:** Yixin Zhang, Jinhui Hu, Juan Liu, Yu Ma, Zhaojun Pan

**Affiliations:** Neonatal Medical Center, Affiliated Hospital of Yang Zhou University Medical College, Huai‘an Maternal and Child Health Care Center, Huai'an, China

**Keywords:** acute kidney injury, continuous renal replacement therapy, multiple organ dysfunction, neonatal shock, perinatal asphyxia, very low birth weight infant

## Abstract

**Introduction:**

Very low birth weight (VLBW) preterm infants are particularly vulnerable to acute kidney injury (AKI) because of immature renal function and limited physiological reserve. However, the use of continuous renal replacement therapy (CRRT) in this population remains technically demanding and clinically challenging. We report a case of successful early CRRT implementation for severe AKI secondary to perinatal asphyxia in a VLBW infant, together with a review of the relevant literature.

**Case presentation:**

A female infant born at 31+6 weeks of gestation with a birth weight of 1,470 g was admitted to the neonatal intensive care unit (NICU) following severe perinatal asphyxia. Shortly after admission, she developed persistent oliguria, progressive metabolic acidosis, azotemia, and fluid overload, consistent with stage 3 AKI. Despite comprehensive supportive management, including mechanical ventilation, vasoactive support, and correction of electrolyte and acid–base disturbances, renal function and hemodynamic status continued to deteriorate. CRRT in continuous venovenous hemodiafiltration (CVVHDF) mode was initiated approximately 20 h after birth using a low initial blood flow rate and a gradual individualized ultrafiltration strategy. After 37 h of treatment, urine output increased from 0.11 to 3.9 mL/(kg·h), serum creatinine decreased from 118.20 to 55.20 μmol/L, and blood urea nitrogen decreased from 12.27 to 5.26 mmol/L, accompanied by marked improvement in metabolic status and circulatory stability. The infant was subsequently weaned from CRRT and invasive mechanical ventilation and was discharged in stable condition.

**Conclusion:**

In VLBW infants with AKI, early and graduated CRRT intervention may contribute to maintaining hemodynamic stability and facilitating organ function recovery. Meticulous peri-CRRT management is essential to ensure treatment safety.

## 1 Introduction

Neonatal acute kidney injury (AKI) is a common and serious complication in the NICU, with an incidence of 18%−40% among very low birth weight (VLBW, < 1,500 g) and extremely low birth weight (ELBW, < 1,000 g) preterm infants. It is associated with prolonged mechanical ventilation, increased in-hospital mortality, and an elevated risk of chronic kidney disease in later life (1, 2). The nephrons in these infants are immature, with high renal vascular resistance and low creatinine clearance. Renal perfusion is readily influenced by mean arterial pressure and tissue oxygenation, rendering these infants susceptible to acute tubular necrosis and medullary stress injury under conditions of ischemia, hypoxia, or reduced perfusion (2, 3).

Severe perinatal asphyxia is an important and potentially modifiable trigger for AKI in VLBW/ELBW preterm infants. Profound hypoxia and acidosis can simultaneously induce myocardial depression, loss of peripheral vascular tone with capillary leak, and reduced circulating volume, resulting in mixed shock with multiorgan hypoperfusion (3, 4). Hypoperfusion, reperfusion injury, systemic inflammation, and microcirculatory dysfunction drive the progressive deterioration of tubular injury and create a vicious cycle; furthermore, fluid overload itself is an independent risk factor for mortality and organ dysfunction (5, 6).

When conventional supportive measures fail to correct persistent oliguria or anuria, refractory metabolic acidosis, or severe fluid overload, renal replacement therapy (RRT) should be considered. Peritoneal dialysis (PD) is technically simple to perform; however, in critically ill infants with hemodynamic instability or those requiring rapid and precise fluid management, its solute clearance and ultrafiltration capacity are often inadequate (7). In contrast, continuous renal replacement therapy (CRRT) enables gradual and continuous solute removal with precise ultrafiltration adjustment, thereby offering superior hemodynamic stability and fluid control, which may make it more suitable for critically ill neonates. Despite these advantages, CRRT in VLBW/ELBW preterm infants remains technically challenging. Major obstacles include difficulty establishing vascular access, the disproportionately large extracorporeal circuit volume relative to circulating blood volume (often requiring blood priming), the need to balance anticoagulation with bleeding risk, and potential complications such as hypotension, hypothermia, and electrolyte imbalance. To date, evidence supporting CRRT for shock-associated AKI secondary to perinatal asphyxia in VLBW/ELBW preterm infants is limited to single-center case reports and small case series, and standardized recommendations regarding initiation timing, vascular access and circuit configuration, or stepwise parameter adjustment are still lacking (8–11). Here, we report the successful application of CRRT in a VLBW preterm infant with severe asphyxia complicated by AKI, and review the relevant literature to provide clinical experience for similar cases.

## 2 Methods

### 2.1 Study subject and ethics review

We retrospectively collected the complete clinical data of one VLBW preterm infant admitted to the NICU of Huai'an Maternal and Child Health Care Center in September 2025, who underwent CRRT for AKI secondary to severe perinatal asphyxia-related shock. Data collected included perinatal history, clinical manifestations, serial blood gas and biochemical analyses, imaging findings, CRRT operational parameters, and clinical outcomes. Key time points were defined as follows: admission (1 h after birth); pre-CRRT (approximately 13 h after birth, representing the most recent systematic blood sampling within approximately 9 h before CRRT initiation); during treatment (approximately 18 h after CRRT initiation); and post-CRRT (approximately 1.5 h after CRRT discontinuation).

This study was approved by the Medical Ethics Committee of Huai'an Maternal and Child Health Care Center (Ethics No.: 2026007), and written informed consent was obtained from the patient's guardians. This case report was prepared in accordance with the CARE (CAse REport) guidelines (12).

### 2.2 Diagnostic criteria for acute kidney injury

AKI was diagnosed and staged according to neonatal-modified KDIGO/nRIFLE criteria, defined as an increase in serum creatinine of ≥26.5 μmol/L (0.3 mg/dL) or ≥1.5-fold from baseline within 7 days and/or urine output < 1.0 mL/(kg·h) for ≥24 hours. Because neonatal serum creatinine during the first 24–72 h partially reflects maternally derived creatinine, interpretation of early postnatal creatinine levels requires caution. Maternal serum creatinine was within the normal adult range. In the present case, the infant exhibited persistent oliguria [0.11 mL/(kg·h)], markedly elevated serum creatinine (118.20 μmol/L), and progressive fluid overload, consistent with stage 3 AKI.

### 2.3 Literature search strategy

To systematically compare this case with previously reported cases, a literature search was conducted in PubMed and Web of Science from database inception to April 2026. The search strategy combined MeSH terms and free-text words: (“continuous renal replacement therapy” OR “CRRT” OR “continuous kidney replacement therapy” OR “CVVHDF” OR “CVVH”) AND (“neonate” OR “newborn” OR “preterm infant” OR “premature infant” OR “very low birth weight” OR “VLBW” OR “extremely low birth weight” OR “ELBW”) AND (“acute kidney injury” OR “AKI” OR “acute renal failure” OR “oliguria”).

Inclusion criteria were: (1) full text available in English; (2) study population comprised neonates and/or preterm/VLBW/ELBW infants (mixed pediatric cohorts extending to older infants were excluded); and (3) clinical application or technical management of CRRT for AKI was reported. Conference abstracts, reviews without original data, and duplicate reports were excluded, and reference lists of included articles were manually searched. Screening and data extraction were performed independently by two reviewers, with discrepancies resolved by a senior author. As this study constitutes a single case report combined with a qualitative literature review rather than a systematic review or meta-analysis, no formal methodological quality scoring of included studies was performed.

## 3 Case presentation

The patient was a female infant born at 31^+6^ weeks of gestation to a G2P2 mother, with a birth weight of 1,470 g. She was delivered by cesarean section because of a scarred uterus, monochorionic diamniotic twin pregnancy complicated by twin-to-twin transfusion syndrome (TTTS), polyhydramnios, threatened preterm labor, and fetal distress, and was the smaller of the two twins. The co-twin, a female infant weighing 1,630 g, had Apgar scores of 6, 7, and 9 at 1, 5, and 10 min, respectively, and remained relatively stable after birth without significant oliguria, metabolic acidosis, or evidence of AKI. In contrast, the present infant had Apgar scores of 1, 2, and 5 at 1, 5, and 10 min, respectively. At birth, the infant was apneic with a heart rate of approximately 40 beats/min. She was immediately intubated and received positive-pressure ventilation, chest compressions, fluid resuscitation, and one intravenous dose of epinephrine (10 μg/kg) via an umbilical venous catheter. Her heart rate recovered to >100 beats/min within approximately 8 min, but spontaneous respiration did not resume. She was subsequently transferred to the NICU with diagnoses of severe perinatal asphyxia and very low birth weight.

On admission (1 h after birth), the infant had a temperature of 33.2 °C, a heart rate of 55 beats per min, a respiratory rate of 22 breaths per min (on mechanical ventilation), and a blood pressure of 42/26 mmHg. She was poorly responsive, with mottled and ashen skin and a capillary refill time of approximately 5 s, indicating severe hypothermia combined with poor perfusion. Arterial blood gas analysis revealed severe metabolic acidosis with insufficient respiratory compensation: pH 7.09, PaCO_2_ 22.7 mmHg, HCO_3_^−^ 7.20 mmol/L, base excess −22.00 mmol/L, anion gap 24.20 mmol/L, and lactate 16.00 mmol/L (see Table 1). These parameters indicated the presence of severe mixed shock (with cardiogenic, distributive, and hypovolemic components) accompanied by significant tissue hypoperfusion and an elevated anaerobic metabolic burden. In addition, blood glucose was 8.20 mmol/L and ionized calcium was 1.05 mmol/L, suggesting stress-related hyperglycemia and electrolyte imbalance.

**Table 1:** Dynamic changes of clinical and laboratory parameters during the peri-CRRT period.

Parameter (Unit)	Reference range	Admission (1 h after birth)	Pre-CRRT (~13 h after birth)	During CRRT (~18 h after initiation)	Post-CRRT (~1.5 h after discontinuation)
Clinical features and fluid management					
Urine output [mL/(kg·h)]	—	—	0.11↓	~1.90↓	3.90
Daily fluid intake (mL/day)^a^	—	—	188.5	223.16	330.78
Daily urine output (mL/day)^a^	—	—	5.00↓	67.80	143.00
Net ultrafiltration rate (mL/h)	—	—	0	3.00–4.00	—
Epinephrine [μg/(kg·min)]	—	—	0.25	0.25 → 0.15	0.15
Blood gas analysis					
pH	7.35–7.45	7.09↓*↓↓*	7.33↓	7.17↓*↓↓*	7.35
PaCO_2_ (mmHg)	35.00–48.00	22.70↓	31.80↓	32.70↓	31.10↓
PaO_2_ (mmHg)	83.00–108.00	68.50↓	61.50↓	64.30↓	179.00↑
HCO_3_^−^ (mmol/L)	18.00–23.00	7.20↓	16.80↓	11.80↓	17.20↓
Base excess (mmol/L)	−2.00–+3.00	−22.00↓	−8.40↓	−15.70↓	−7.30↓
Lactate (mmol/L)	0.50–2.20	16.00↑	9.20↑	1.40	2.40↑
Anion gap (mmol/L)	8.00–16.00	24.20↑	22.70↑	7.50↓	12.50
Electrolytes					
K^+^ (mmol/L)	4.20–5.90	3.50	2.82↓	4.85	4.10
Na^+^ (mmol/L)	135–150	139.0	133.50↓	139.40	144.0
Cl^−^ (mmol/L)	100–116	108.0	98.60↓	116.50↑	115.0
Serum Ca^2+^ (mmol/L)	2.10–2.80	—	1.80↓	1.72↓	—
Ionized Ca^2+^ (mmol/L)	1.12–1.32	1.05↓	—	0.53↓	0.73↓
Mg^2+^ (mmol/L)	0.65–1.25	—	0.79	0.90	—
Inorganic phosphate (mmol/L)	1.60–2.51	—	1.73↑	—	—
Renal function					
Blood urea nitrogen (mmol/L)	0.80–5.30	—	12.27↑	—	5.26
Serum creatinine (μmol/L)	13.00–33.00	—	118.20↑	—	55.20↑
Uric acid (μmol/L)	184.00–464.00	—	547.30↑	—	129.30↓
Liver function					
AST (U/L)	21.00–80.00	—	530.00↑	—	—
ALT (U/L)	8.00–71.00	—	95.00↑	—	—
GGT (U/L)	7.00–32.00	—	270.00↑	—	—
ALP (U/L)	98.00–532.00	—	123.00	—	—
Total bile acids (μmol/L)	0–10.00	—	24.30↑	—	—
Total bilirubin (μmol/L)	5.00–21.00	—	59.20↑	—	141.00↑
Direct bilirubin (μmol/L)	0–6.80	—	9.80↑	—	—
Indirect bilirubin (μmol/L)	0–16.60	—	49.40↑	—	—
Total protein (g/L)	49.00–71.00	—	33.9↓	—	—
Albumin (g/L)	35.00–50.00	—	22.1↓	—	—
Cardiac Enzymes					
CK (U/L)	34.00–145.00	—	1,009.00↑	—	—
CK-MB (U/L)	0–25.00	—	1,231.10↑	—	—
LDH (U/L)	120.00–300.00	—	2,023.00↑	—	—
α-HBDH (U/L)	72.00–182.00	—	1,086.60↑	—	—
Coagulation					
PT (s)	9.40–12.50	—	15.00↑	12.40↑	11.70
INR	0.80–1.20	—	1.38↑	1.13	1.07
APTT (s)	25.10–36.50	—	38.70↑	88.70↑*↑↑*	37.90↑
Fibrinogen (g/L)	2.38–4.98	—	1.28↓	0.89↓	1.43↓^b^
Thrombin time (s)	10.30–16.60	—	18.50↑	31.20↑	19.00↑
D-dimer (mg/L)	0.00–0.50	—	27.19↑	96.26↑	15.14↑
FDP (μg/mL)	0.00–5.00	—	68.43↑	209.40↑	31.91↑
Metabolic					
Blood glucose (mmol/L)	3.30–5.30	8.20↑	22.36↑	7.20↑	4.10

↑/↓ indicates above/below the reference range; ↑*↑↑*/↓*↓↓* indicates a critical value; — indicates not measured or not applicable.

^a^ Daily fluid intake and urine output are accumulated per calendar day (24 h): 25 Sep encompasses the pre-CRRT period and approximately 4 h post-initiation; 27 Sep encompasses approximately 9.5 h pre-weaning and 14.5 h post-weaning.

^b^ On the day of CRRT discontinuation, cryoprecipitate was transfused to replenish coagulation factors and provide colloid, in view of persistently low fibrinogen levels and ongoing edema.

ALP, alkaline phosphatase; ALT, alanine aminotransferase; APTT, activated partial thromboplastin time; AST, aspartate aminotransferase; BE, base excess; CK, creatine kinase; CK-MB, creatine kinase-MB isoenzyme; CRRT, continuous renal replacement therapy; CVVHDF, continuous venovenous hemodiafiltration; FDP, fibrinogen degradation products; FIB, fibrinogen; GGT, gamma-glutamyl transferase; α-HBDH, alpha-hydroxybutyrate dehydrogenase; INR, international normalized ratio; LDH, lactate dehydrogenase; PaCO_2_/PaO_2_, arterial partial pressure of carbon dioxide/oxygen; PT, prothrombin time; TT, thrombin time.

Following admission, a series of comprehensive interventions were implemented to stabilize the circulation and correct internal milieu disturbances. Stepwise rewarming using a radiant warmer combined with an incubator was employed to avoid the hemodynamic instability associated with rapid temperature elevation. High-frequency oscillatory ventilation (HFOV) combined with intratracheal surfactant instillation was applied to correct hypoxemia. For hemodynamic support, low-dose epinephrine at 0.25 μg/(kg·min) combined with stepwise fluid resuscitation was used to maintain circulatory stability. Empirical broad-spectrum antimicrobial therapy was initiated concurrently, and microbiological specimens were obtained to identify potential pathogens. Throughout this period, fluid balance, blood gas, electrolytes, coagulation parameters, and hepatorenal function were closely and dynamically monitored to inform real-time clinical decision-making. Despite these comprehensive interventions, total urine output during the first 9 h of admission remained only approximately 2 mL [0.11 mL/(kg·h)]. At approximately 13 h after birth, serum creatinine had risen to 118.20 μmol/L, blood urea nitrogen was 12.27 mmol/L, and uric acid was 547.30 μmol/L. Arterial blood gas showed pH 7.33, HCO_3_^−^ 16.80 mmol/L, and base excess −8.4 mmol/L, indicating ongoing progression of AKI (KDIGO stage 3) following severe asphyxia, with conventional supportive measures unable to reverse the increasing fluid load and internal milieu disturbances (Table 1).

In light of the clinical trajectory, a rapid multidisciplinary consultation (involving neonatology, nephrology, ultrasonography, and nursing) was convened to comprehensively evaluate the timing of CRRT initiation. The infant had persistent anuria or severe oliguria with markedly elevated serum creatinine and azotemia, progressive fluid overload despite meticulous fluid management throughout the admission period, metabolic acidosis that could not be corrected by bicarbonate administration alone, and ongoing dependence on relatively high doses of vasoactive agents for circulatory support. Given that oxygenation was relatively stable, the internal milieu had been partially corrected, and the infant was considered able to tolerate the procedure, the team decided to initiate CRRT in CVVHDF mode at approximately 20 h after birth (25 September 2025, 20:16), combining convection and diffusion to enhance clearance of small to medium molecular weight toxins. A 6.5-Fr, 5-cm double-lumen catheter was inserted into the right internal jugular vein under ultrasound guidance. CRRT was performed using the Asahi Kasei PLASUTO-Σ system equipped with a CRRT-CSGNL1 pediatric filter. The extracorporeal circuit was primed with packed red blood cells and actively warmed throughout treatment to minimize hemodynamic instability and heat loss. Specific CRRT operational parameters are detailed in Table 2.

**Table 2:** Operational parameters of CRRT.

Parameter	Setting/Value
Treatment modality	CVVHDF (continuous venovenous hemodiafiltration)
Initiation/discontinuation time	25 Sep 2025, 20:16/27 Sep 2025, 09:24
Total treatment duration	37 h
Blood flow rate	Started at 6 mL/min; gradually increased to 8 mL/min
Dialysate flow rate	100 mL/h
Replacement fluid flow rate	Started at 20 mL/h; gradually increased to 30 mL/h
Net ultrafiltration rate	Started at 0 mL/h; progressively adjusted to 3–4 mL/h based on fluid balance
Anticoagulation	Heparin 1 mL/h (≥10 IU/h); dose titrated with APTT monitoring every 4–6 h
Cumulative filtration volume	4.09 L
Cumulative replacement fluid volume	0.75 L
Cumulative dialysate volume	3.18 L
Cumulative net fluid removal	0.158 L

Parameters were dynamically adjusted throughout treatment based on hemodynamic status, hourly fluid balance, electrolyte levels, and coagulation monitoring. Blood flow rate was started at the minimum setting and net ultrafiltration was initiated at zero; both were gradually increased after circulatory stabilization was confirmed. Anticoagulant dose was individually titrated with APTT monitoring at 4–6 h intervals. Criteria for CRRT discontinuation: recovery of spontaneous urine output (approximately 3.9 mL/(kg·h) over the preceding 24 h), reduction in edema, and stabilization of the internal milieu with no new organ support requirements.

APTT, activated partial thromboplastin time; CRRT, continuous renal replacement therapy; CVVHDF, continuous venovenous hemodiafiltration.

After initiation, CRRT was managed according to a “low start, gradual titration, multiparameter monitoring” principle. Blood flow rate was started at 6 mL/min and incrementally increased to 8 mL/min. Dialysate flow rate was set at 100 mL/h, and replacement fluid flow rate was gradually increased from 20 mL/h to 30 mL/h. Net fluid removal was initiated at 0 mL/h and progressively increased to 3–4 mL/h based on continuous hourly fluid balance, central venous pressure, capillary refill time, heart rate, blood pressure, and peripheral oxygen saturation. Anticoagulation was achieved with continuous low-dose heparin infusion at 1 mL/h (approximately 10 IU/h), titrated with activated partial thromboplastin time (APTT) monitoring at 4–6 h intervals. Blood gas, electrolytes, blood glucose, lactate, and coagulation parameters were reassessed every 4–8 h, and parameters were further adjusted based on dynamic trends. During the treatment course, vasoactive drug doses were progressively reduced in accordance with hemodynamic improvement [epinephrine: 0.25 to 0.15 μg/(kg·min)].

**Figure 1:**
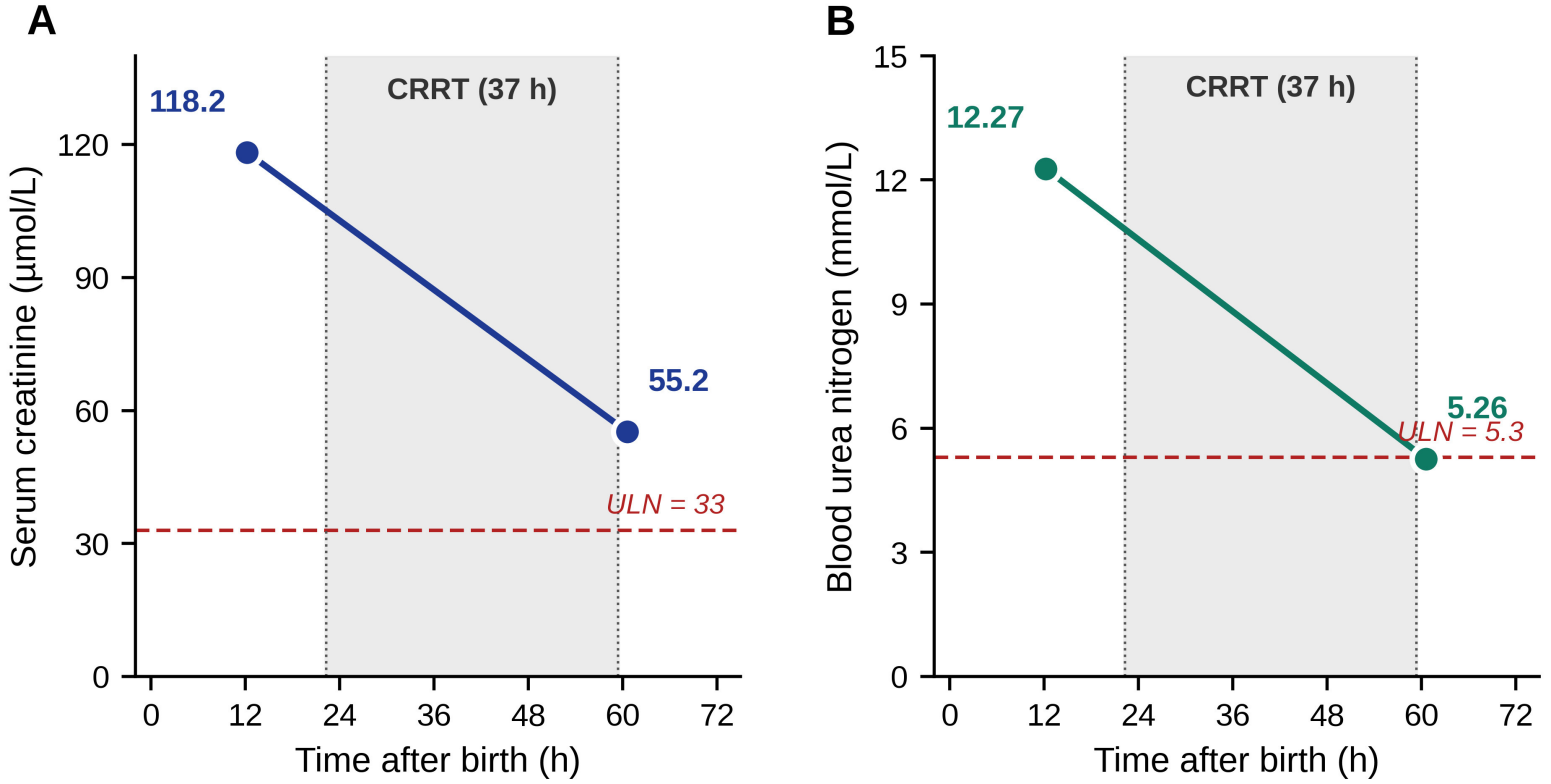
**(A, B)** The changes in blood urea nitrogen and creatinine levels before and after CRRT. CRRT, continuous renal replacement therapy.

CRRT was maintained for a total of 37 h (25 September 2025, 20:16 to 27 September 2025, 09:24), with a cumulative filtration volume of 4.09 L, replacement fluid volume of 0.75 L, dialysate volume of 3.18 L, and net fluid removal of 0.158 L (158 mL). At the time of CRRT discontinuation (approximately 1.5 h after cessation), urine output had recovered to 3.9 mL/(kg·h). Serum creatinine decreased from 118.20 μmol/L to 55.20 μmol/L, blood urea nitrogen from 12.27 mmol/L to 5.26 mmol/L, and uric acid from 547.30 μmol/L to 129.30 μmol/L. Arterial blood gas showed pH 7.35, base excess −7.3 mmol/L, and lactate 2.4 mmol/L. Electrolytes (K^+^ 4.10 mmol/L, Na^+^ 144.0 mmol/L) and coagulation indices (PT 11.7 s, APTT 37.9 s, INR 1.07) were all significantly improved. D-dimer and fibrinogen degradation products (FDP) showed a notable decline, reflecting an improving trend in consumptive coagulopathy (Table 1). Criteria for CRRT discontinuation were: recovery of spontaneous urine output (approximately 3.9 mL/(kg·h) over the preceding 24 h), reduction in edema, stabilization of the internal milieu, and no new organ support requirements. The changes in blood urea nitrogen and creatinine levels before and after CRRT in the patient are detailed in Figure 1.

Following CRRT discontinuation, the infant was transitioned to non-invasive ventilatory support, vasoactive drugs were gradually withdrawn, and comprehensive management continued, including antimicrobial therapy, establishment of enteral feeding, phototherapy for jaundice, and neurodevelopmental assessment. Serial cranial ultrasonography and pre-discharge brain MRI revealed no evidence of intraventricular hemorrhage, periventricular leukomalacia, or hypoxic–ischemic brain injury. No catheter-related bloodstream infection, major hemorrhage, recurrent hypothermia, hemodynamic instability-related complications, or pressure injuries occurred during the hospital stay. After 35 days of hospitalization, the infant achieved clinical stability with a normalized internal milieu, complete recovery of renal function, and intact neurological reflexes, and was discharged in good condition. The infant has remained under outpatient follow-up since discharge. At the most recent evaluation at 6 months of age, renal function was normal, and physical growth and neurodevelopmental assessments were age-appropriate, as evaluated using the Gesell Developmental Schedules and the Hammersmith Infant Neurological Examination.

## 4 Discussion

On admission, this infant exhibited a classic presentation of severe mixed neonatal shock, a pathophysiological state closely linked to the development of AKI (3, 4). Ischemia and hypoxia directly cause ATP depletion and apoptosis in renal tubular epithelial cells; during the subsequent reperfusion phase, oxidative stress, endothelial glycocalyx shedding, and activation of complement and inflammatory pathways collectively amplify tubulointerstitial injury. Concurrently, hypothermia induces marked renal vasoconstriction and a decline in glomerular filtration rate, further accelerating the progression of renal injury. Interpretation of serum creatinine in the early neonatal period requires caution because levels during the first 24–72 h partly reflect maternally derived creatinine. In the present case, however, persistent severe oliguria, progressive fluid overload, metabolic derangement, and markedly elevated serum creatinine strongly supported true neonatal AKI. Critically, once AKI is established, it tends to become self-reinforcing through a vicious cycle: hypoperfusion leads to renal injury, which causes oliguria and fluid retention, resulting in volume overload that reduces cardiopulmonary compliance and in turn worsens perfusion. Multicenter pediatric studies by Sutherland et al. (5) and Selewski et al. (6) both demonstrated that cumulative fluid overload of 10%−20% or greater is independently associated with mortality in critically ill pediatric patients. Therefore, timely interruption of this vicious cycle carries important prognostic implications for low birth weight preterm infants with severe asphyxia-related shock and AKI.

Regarding the optimal timing of CRRT initiation in neonates and low birth weight preterm infants, no consensus currently exists. Prior reports have generally deferred CRRT until overt fluid overload, multiorgan dysfunction, or persistent anuria lasting more than 24–48 h had developed (13). A distinctive feature of the present case was the approach of early recognition and early intervention: the care team identified a trend of persistent oliguria with rapidly rising serum creatinine as early as 9 h after birth, and rather than awaiting the development of fluid retention or multiorgan failure, initiated CRRT at approximately 20 h after birth once acid-base disturbances had been partially corrected and the circulatory state had improved. The rationale for early initiation includes a fluid overload that has not yet reached an irreversible level, a smaller ultrafiltration target with correspondingly less circulatory perturbation, a more manageable hemodynamic state that allows the infant to tolerate mild blood pressure fluctuations at initiation, and earlier clearance of uremic toxins and inflammatory mediators that may help break the hypoperfusion-renal injury-fluid retention cycle. Recent pediatric CRRT studies have also shown that early initiation is associated with lower fluid overload and better survival outcomes (5, 6, 13). With regard to the specific management strategy after initiation, this case employed a low start, gradual titration approach: blood flow rate was started at 6 mL/min, ultrafiltration rate was started at zero, and anticoagulation was achieved with continuous low-dose heparin titration. This strategy substantially reduces the risk of hemodynamic instability during circuit priming and CRRT initiation, and is particularly well suited for low birth weight preterm infants in whom the extracorporeal circuit volume represents a high proportion of the circulating blood volume (9, 11). Furthermore, multiparameter monitoring every 4–8 h allowed parameters to be continuously and individually adjusted based on actual physiological responses rather than empirically prescribed at preset doses.

The technical risks of CRRT in VLBW preterm infants are substantially greater than in term infants or older children. Drawing on the clinical experience gained in this case and on the existing literature, several key considerations can be identified for peri-CRRT management. Vascular access should be established under bedside ultrasound guidance using an appropriately sized double-lumen catheter, balancing the difficulty of insertion, achievable blood flow rate, and hemodynamic impact. In the present case, no catheter-related hemorrhage, thrombosis, or infection occurred, consistent with the experience reported by Wu et al. in low birth weight infants (11). Circuit and priming strategies should be individualized to minimize the hemodynamic and thermal perturbations associated with circuit volume and temperature. Regarding anticoagulation, low-dose continuous heparin infusion titrated to APTT was used. During treatment, APTT transiently increased to 88.7 s, prompting temporary suspension of heparin followed by dose reduction after coagulation parameters improved. No clinical bleeding or circuit thrombosis occurred, highlighting the importance of close coagulation monitoring and individualized anticoagulation adjustment, particularly in VLBW/ELBW infants.

Although regional citrate anticoagulation (RCA) has been reported to be feasible in neonates (14), its use requires dedicated equipment and stringent calcium monitoring. Temperature maintenance should be ensured through incubators, warming blankets, and circuit warming devices to sustain a core temperature of at least 36.5 °C. Blood gas and biochemical parameters should be reassessed every 4–8 h to prevent rebound cerebral or cardiac injury from overly rapid correction of electrolyte or acid-base disturbances. Infection control and skin care are equally important and encompass standardized catheter-site management, pressure injury prevention, adherence to disinfection protocols, and implementation of regular multidisciplinary ward rounds. Through these comprehensive measures, effective CRRT can be conducted while preserving patient safety.

A total of 14 published reports describing CRRT for AKI in neonates and low birth weight preterm infants were identified (8–11, 13–22). The series most directly comparable to the present case was that of Cheng et al. (9), who reported five ELBW preterm infants (birth weight 450–960 g) from a tertiary NICU in China; a detailed comparison with the present case is provided in Table 3. That series employed CVVHDF with blood priming and citrate anticoagulation; while all infants were successfully initiated on CRRT, the 28-day survival rate was only 2 out of 5, and both surviving infants ultimately had care withdrawn due to severe neurological sequelae. Yang et al. (10) reported one ELBW infant (birth weight 0.89 kg) with sepsis-induced capillary leak syndrome who was successfully treated with 82 h of CVVHDF and discharged. A multicenter study by Garzotto et al. (18) indicated that survival in neonates receiving CRRT was significantly associated with body weight, with a survival rate of 31% in those weighing less than 3 kg compared with 69% in those weighing more than 3 kg. In contrast to previous reports, which largely involved sepsis-, NEC-, or hyperkalemia-related AKI treated after overt fluid overload had developed, the present case involved early CRRT initiation for asphyxia-related AKI guided by dynamic clinical deterioration. Distinctive features included VLBW status, severe perinatal asphyxia with mixed shock as the primary AKI trigger, CRRT initiation at approximately 20 h after birth, and successful discontinuation after a single 37-hour session using a gradual low-parameter titration strategy. Compared with prior reports, oliguria duration before CRRT was shorter and intervention was initiated earlier. These findings suggest that, in selected VLBW infants with potentially reversible AKI, early carefully titrated CRRT may be feasible and may facilitate short-term renal and hemodynamic recovery.

**Table 3:** Head-to-head comparison of the present case with the ELBW case series reported by Cheng et al. (9).

Parameter	Present case (*n* = 1)	Cheng et al. [9] (*n* = 5)
Patient characteristics		
Gestational age (weeks)	31+6	23^+1^-28
Birth weight (g)	1,470	450–960
Weight at CRRT initiation (g)	1,470	700–1,090
Primary diagnosis	Severe perinatal asphyxia; mixed shock (cardiogenic + distributive + hypovolemic)	Sepsis, NEC, hyperkalemia, anuria
AKI profile at CRRT initiation		
AKI etiology	Asphyxia-related renal hypoperfusion	Sepsis/NEC/multiorgan dysfunction
AKI stage (KDIGO)	Stage 3	Not individually specified
Pre-CRRT serum creatinine (μmol/L)	118.20	Not individually reported
Pre-CRRT urine output	0.11 mL/(kg·h)	Oliguria/anuria in all
CRRT Prescription		
Modality	CVVHDF	CVVHDF
Timing of initiation	~20 h after birth	As early as day 3 of life
Vascular access	6.5-Fr, 5 cm dual-lumen catheter via right internal jugular vein; ultrasound-guided	5.0-Fr dual-lumen catheter; IJV or femoral; ultrasound-guided
Initial blood flow rate (mL/min)	6 → 8	1 → titrated to tolerance
Anticoagulation	Low-dose heparin (≈10 IU/h); APTT-guided	RCA (sodium citrate; target iCa^2+^ < 0.4 mmol/L)
Treatment response and outcome		
CRRT duration (h)	37 (single session)	≤ 65 cumulative; maximum single session 12 h
Post-CRRT serum creatinine (μmol/L)	55.20	Maximum clearance rate 16.84 μmol/(L·h) at 6 h
Urine output recovery	3.9 mL/(kg·h)	Improved in all 5 patients
Acid-base correction	pH 7.09 → 7.35; BE −22 → −7.3; lactate 16 → 2.4 mmol/L	pH normalized in all
Complications	APTT transiently elevated to 88.7 s (no bleeding); no infection, hypothermia, or hemodynamic instability	Hypotension in 1; catheter occlusion in 1; IVH/PVL in 2 (pre-existing)
Survival/outcome	Survived; discharged on day 35; renal function fully recovered; normal physical and neurodevelopmental assessment at 6-month follow-up	28-day survival 2/5; both survivors had care withdrawn due to severe neurological sequelae

APTT, activated partial thromboplastin time; BE, base excess; CVC, central venous catheter; CVVHDF, continuous venovenous hemodiafiltration; ECV, extracorporeal volume; iCa^2+^, ionized calcium; IJV, internal jugular vein; IVH, intraventricular hemorrhage; NEC, necrotizing enterocolitis; PVL, periventricular leukomalacia; RCA, regional citrate anticoagulation.

In VLBW preterm infants with severe perinatal asphyxia complicated by mixed shock and AKI, the clinical course should be interpreted within the context of the hypoperfusion–renal injury–fluid overload cycle rather than as isolated renal dysfunction. When progressive oliguria, worsening renal function, metabolic derangement, and fluid overload persist despite conventional supportive therapy, CRRT may be considered after initial hemodynamic stabilization using a low-start, gradually titrated, individualized strategy. In the present case, early carefully titrated CRRT was temporally associated with improvement in solute clearance, fluid balance, acid–base status, and hemodynamic stabilization. Standardized peri-CRRT management and close multidisciplinary collaboration were important for treatment safety and procedural feasibility. Nevertheless, this report describes a single infant at the relatively favorable end of the VLBW spectrum (1,470 g) with a potentially reversible AKI etiology, and the findings may not be generalizable to ELBW infants or other high-risk neonatal populations. In addition, mechanistic evaluation of inflammatory mediators and renal injury biomarkers was not performed. Further multicenter prospective studies are needed to clarify the optimal timing of CRRT initiation, parameter adjustment strategies, anticoagulation protocols, and long-term outcomes in this population.

## Data Availability

The original contributions presented in the study are included in the article/supplementary material, further inquiries can be directed to the corresponding authors.
